# Normative Reference Values and Cut‐Off Points for Nutritional Ultrasound: The VALORES Study

**DOI:** 10.1002/jcsm.70344

**Published:** 2026-08-19

**Authors:** Carolina Moreno‐Torres‐Taboada, José M. Romero‐Márquez, María García‐Olivares, María Novo‐Rodríguez, Rocío Fernández‐Jiménez, Isabel Vegas‐Aguilar, Pedro Pablo García‐Luna, Silvia García‐Rey, José Manuel García‐Almeida, Gabriel Olveira, for the VALORES Group, Martín López‐de‐la‐Torre‐Casares, Araceli Muñoz‐Garach, Cristina Novo‐Rodríguez, Juan Manuel Guardia‐Baena, Marina Padial‐Barranco, Nuria Porras‐Pérez, Gemma Rojo‐Martínez, María del Carmen Roque‐Cuéllar, Andrés Jiménez‐Sánchez, Antonio Jesús Martínez‐Ortega

**Affiliations:** ^1^ Department of Endocrinology and Nutrition Regional University Hospital of Málaga Málaga Spain; ^2^ Department of Medicine and Dermatology, Faculty of Medicine University of Malaga Málaga Spain; ^3^ Department of Endocrinology and Nutrition Virgen de las Nieves University Hospital Granada Spain; ^4^ Foundation for Biosanitary Research of Eastern Andalusia ‐ Alejandro Otero (FIBAO) Granada Spain; ^5^ Faculty of Health Sciences. Department of Nutrition and Sports Sciences International University of La Rioja Logroño Spain; ^6^ IBIMA BIONAND Platform Biomedical Research Institute of Málaga Málaga Spain; ^7^ Department of Endocrinology and Nutrition QuirónSalud Málaga Hospital Málaga Spain; ^8^ Department of Endocrinology and Nutrition Virgen de la Victoria University Hospital Málaga Spain; ^9^ Nutrition Unit, Endocrinology and Nutrition Department Virgen del Rocío University Hospital Sevilla Spain; ^10^ Network Biomedical Research Center Physiopathology of Obesity and Nutrition (CiberOBN) Carlos III Health Institute Madrid Spain; ^11^ Biomedical Research Networking Center in Diabetes and Associated Metabolic Disorders (CIBERDEM) Instituto de Salud Carlos III Málaga Spain

**Keywords:** body composition, malnutrition, muscle ultrasound, nutritional ultrasound, reference values, sarcopenia

## Abstract

**Background:**

Nutritional ultrasound (NU) enables direct assessment of skeletal muscle and adipose tissue, yet population‐based reference values and cut‐offs remain scarce. We aimed to establish age‐ and sex‐specific normative values for rectus femoris (RF) and abdominal adipose tissue NU measurements in apparently healthy adults.

**Methods:**

Multicentre cross‐sectional study of 1789 healthy blood donors (842 women, 947 men), aged 18–80 years (mean 41.5 ± 14.9). Standardised NU assessed RF cross‐sectional area (CSA), circumference, transverse (X) and anteroposterior (Y) axes, and abdominal and thigh adipose tissue. Anthropometry, bioelectrical impedance analysis (BIA) and handgrip strength (HGS) were obtained. Age‐ and sex‐specific percentiles were generated across predefined age groups (18–30, 31–45, 46–60 and > 60 years). T‐scores for RF parameters were derived from a young reference subgroup aged 18–45 years (*n* = 1001). Associations with sex, age, body mass index and physical activity were analysed.

**Results:**

All NU parameters differed significantly by sex (all *p* < 0.001). Median RF‐CSA was 4.60 cm^2^ (IQR 3.83–5.58) in women and 6.77 cm^2^ (5.55–7.91) in men; median RF‐Y axis at rest was 1.56 cm (1.33–1.80) and 1.91 cm (1.65–2.17), and during contraction 1.97 cm (1.71–2.24) and 2.47 cm (2.15–2.78), respectively. Median total abdominal subcutaneous adipose tissue (T‐SAT) was 1.77 cm (1.16–2.39) in women and 1.65 cm (1.13–2.22) in men. RF measurements correlated moderately–strongly with BIA‐derived muscle mass (*r* = 0.45–0.65) and HGS (*r* = 0.49–0.58). T‐SAT correlated with BIA fat mass and waist circumference (*r* = 0.45–0.65), whereas visceral adipose tissue showed weaker associations (*r* ≈ 0.33). RF‐CSA and RF‐Y axis increased across physical‐activity categories (*p* < 0.05). Absolute RF values did not differ by BMI; however, when indexed to body weight, participants with BMI > 25 kg/m^2^ had lower RF‐CSA and RF‐Y values (all *p* < 0.001). RF parameters followed a normal distribution, whereas adipose tissue measures did not. In the young reference group (18–45 years), mean ± SD RF‐Y axis was 1.68 ± 0.33 cm in women and 2.05 ± 0.37 cm in men. When applying T‐score criteria, the −2 standard deviation cut‐off identified 1.4%–11.7% of adults aged ≥45 years as having low muscle mass.

**Conclusions:**

This study provides the first age‐ and sex‐specific population‐based reference values for nutritional ultrasound measurements in an apparently healthy adult Spanish population. These findings support the validity, clinical applicability, and standardisation of nutritional ultrasound for the assessment of muscle mass, with potential utility in the diagnosis of sarcopenia and malnutrition.

## 1 Introduction

Disease‐related malnutrition and sarcopenia are both highly prevalent conditions and carry significant clinical consequences, including higher morbidity and mortality, functional deterioration, poorer quality of life and increased healthcare use [1, 2, 3].

Nutritional ultrasound (NU) has become an increasingly adopted method for the direct assessment of body composition. It provides anatomical measurements of muscle architecture and abdominal adipose tissue distribution with a technique that is non‐invasive, radiation‐free and readily available, making it well suited to routine clinical evaluation [4, 5, 6].

Despite its expanding use, reference values for NU in healthy adults are still lacking. Variation in anatomical landmarks, probe handling, insonation angle and measurement protocols has limited comparability across studies. Recent methodological frameworks in body composition research emphasise the importance of precise terminology and structured conceptual models. Prado and colleagues propose five levels of analysis (atomic, molecular, cellular, tissue–organ and whole‐body), each requiring its own set of definitions and measurement models [7]. Within this framework, NU warrants standardised protocols that clearly specify the anatomical landmark, transducer technique and measurement conditions [4, 5].

The absence of robust reference values may explain why current diagnostic criteria, such as GLIM, and the emerging definitions of sarcopenia and sarcopenic obesity have not yet incorporated NU as a primary method [1, 2, 8]. The 2025 GLIM update explicitly highlights the need for cut‐off points adjusted for sex, age, ethnicity and the measurement technique. Similarly, consensus statements on sarcopenic obesity call for further validation of existing indices, including their normalisation for height [3, 8].

In Spain, widespread adoption of harmonised NU protocols across some clinical nutrition units has facilitated more consistent research outputs [9, 10, 11, 12, 13, 14]. NU has shown good agreement with established techniques—including dual‐energy X‐ray absorptiometry (DXA), computed tomography (CT) and bioelectrical impedance analysis (BIA)—and is responsive to nutritional and exercise interventions [9, 12, 14, 15, 16, 17, 18, 19, 20]. Measurements of the rectus femoris (RF) correlate with functional capacity and clinical prognosis, while preperitoneal adipose thickness relates to cardiometabolic risk [10, 11, 20, 21, 22, 23].

Reference values may be derived from population‐based sampling or from sufficiently large convenience samples of individuals without overt disease. Blood donation centres provide an efficient setting for recruiting adults with stable health status and no clinical conditions likely to affect body composition.

The aim of this study was to establish NU reference values in apparently healthy adults, stratified by sex and age, to support clinical interpretation and contribute to the methodological standardisation of the technique.

## 2 Methods

### 2.1 Study Design

This multicentre cross‐sectional study was conducted between September 2023 and February 2025 in the Transfusion, Tissue and Cell Centers of Málaga, Sevilla and Granada. All assessments were integrated seamlessly into the routine blood donation workflow without altering standard procedures.

The target population comprised adults from the general population who attended voluntarily to donate blood. Eligible participants were those who self‐identified as healthy, reported no physician‐diagnosed chronic diseases or regular medication use, and had a body mass index (BMI) between 18.5 and 35 kg/m^2^. Exclusion criteria included acute or recent illness within the previous month, professional athletic activity, unintentional weight change exceeding 5% in the past 3 months, pregnancy or breastfeeding, and the presence of prostheses or other factors likely to interfere with bioelectrical impedance measurements.

Individuals meeting the inclusion criteria were informed about the study and invited to participate. Written informed consent was obtained from all participants, and full data anonymisation was ensured. No biological samples were stored for research purposes. The study adhered to the principles of the Declaration of Helsinki and commenced following approval of the protocol by the Málaga Provincial Research Ethics Committee (CEI) on 31 August 2023.

### 2.2 Questionnaires

Health status and eligibility were determined through structured self‐report complemented by standard pre‐donation screening procedures. All donors underwent the standard pre‐donation assessment in accordance with European Committee on Blood Transfusion [24], including a clinical questionnaire covering medical history, current symptoms, medication use and recent health events; measurement of blood pressure, heart rate and body temperature; and point‐of‐care haemoglobin determination. A brief clinical interview with trained healthcare staff was conducted to confirm eligibility and identify conditions that could contraindicate donation or affect study measurements. Structured questionnaires were then used to collect:

- sociodemographic data: sex, age, marital status, educational attainment, occupation and postcode;
- habitual physical activity, assessed with the IPAQ [25];
- smoking status and alcohol consumption;
- dietary habits using the 14‐item PREDIMED questionnaire [26];
- personal and family history of metabolic diseases.

### 2.3 Morphofunctional Assessment

#### 2.3.1 Anthropometry

Anthropometric measurements followed standardised procedures using calibrated equipment. Weight was measured to the nearest 0.1 kg using a SECA 665 scale (Seca, Germany), and height to the nearest 0.01 m with a Holtain stadiometer (Holtain Ltd., Croswell, United Kingdom), from which BMI was calculated.

#### 2.3.2 Bioelectrical Impedance Analysis (BIA)

BIA was assessed using a phase‐sensitive, single‐frequency analyser (NUTRILAB, Akern Srl, Pontassieve, Italy). The device delivers a 50 kHz alternating sinusoidal current of 400 μA (±0.1%), with a resolution of ±1% for both resistance (Rz) and reactance (Xc), and coefficients of variation below 2%. Measurements followed a standard whole‐body tetrapolar configuration. Participants were examined in the supine position, with the legs abducted approximately 45° from the midline and the arms positioned about 30° from the trunk. Low‐impedance Ag/AgCl electrodes (BIVATRODES, Akern Srl, Florence, Italy) were placed on the dorsal surface of the right hand and on the corresponding foot, with the sensing and injecting areas separated by 5 cm.

The analyser provided raw bioelectrical variables—resistance (R), reactance (Xc), phase angle (PhA), fat‐free mass (FFM), body cell mass (BCM) and skeletal muscle mass (SMM). All assessments were carried out in the supine position following a brief rest period prior to blood donation.

#### 2.3.3 Functional Outcome

Handgrip strength (HSG) was assessed using a JAMAR dynamometer (J.A. Preston Corporation, New York, New York, United States). Participants were seated with the shoulder in adduction and the elbow positioned at 90°, keeping the wrist and forearm of the dominant side in a neutral alignment. Handgrip strength was assessed with the forearm unsupported. Participants were verbally encouraged to perform a maximal contraction for a few seconds, and the peak value was recorded. Three consecutive measurements were taken, with a rest period between attempts, and the mean were used for analysis.

#### 2.3.4 Nutritional Ultrasound (NU)

A detailed NU assessment was carried out following previously published methodology to evaluate muscle and fat mass at the rectus femoris (RF), as well as abdominal adiposity. Ultrasound imaging was performed using a Mindray M9 system (Mindray, Madrid, Spain) equipped with a 10–12 MHz linear probe. Ultrasound measurements were performed directly on the device using the integrated measurement tools, without offline image analysis or external software. Participants were examined in a relaxed supine position with the knee fully extended. Probe pressure was kept to a minimum, ensuring preservation of the gel layer between the probe and the skin in the ultrasound image to avoid tissue compression.

The anatomical landmark for RF measurements was standardised in accordance with the protocol of García‐Almeida et al. [5]. Measurement site was identified at the distal third of the line connecting the anterior superior iliac spine and the superior border of the patella, using a non‐elastic measuring tape. The following parameters were recorded: RF cross‐sectional area (RF‐CSA), RF circumference and the RF X‐ and Y‐axes, representing the transverse and anteroposterior muscle dimensions, respectively. Leg subcutaneous adipose tissue (L‐SAT) was also quantified.

Abdominal ultrasound scans were obtained at the midpoint between the xiphoid process and the umbilicus along the midline. From these images, total subcutaneous abdominal fat (T‐SAT), superficial subcutaneous abdominal fat (S‐SAT) and preperitoneal/visceral adipose tissue (VAT) were measured.

All NU examinations were conducted by dietitians–nutritionists with formal training and prior experience in nutritional ultrasound in both clinical practice and research settings. To minimise intra‐observer variability, each assessment was supervised by a second trained clinician, and measurements were jointly reviewed. High intra‐ and inter‐operator agreement was confirmed, with Pearson correlation coefficients exceeding 0.90 for all operators.

### 2.4 Statistical Analysis

Descriptive statistics were used to summarise quantitative variables (mean, standard deviation, range and population percentiles) and qualitative variables (frequencies and percentages). Age‐group stratification was defined to reflect different stages of adult life and the well‐described trajectory of skeletal muscle across the lifespan, characterised by peak muscle mass in early adulthood, relative stability through midlife, and progressive decline after the fifth decade [27, 28].

Comparisons between two independent groups were performed using Student's *t*‐test when normality assumptions were met, or the Mann–Whitney *U* test otherwise. Welch's correction was applied when variances were unequal. Associations between categorical variables were analysed using the χ^2^ test.

For comparisons involving more than two groups, one‐way ANOVA with Bonferroni‐adjusted post hoc analyses was used for normally distributed variables. When normality assumptions were not fulfilled, the Kruskal–Wallis test was applied, followed by pairwise Mann–Whitney *U* tests with appropriate correction for multiple comparisons.

Correlations between NU variables, anthropometric measurements, HSG and BIA parameters related to muscle and adipose tissue were assessed using Pearson's or Spearman's correlation coefficients, according to data distribution.

Normality was assessed for each NU variable using the Kolmogorov–Smirnov test. When normality criteria were met, reference values for the healthy young adult subgroup (18–45 years) were estimated, and proposed T‐score thresholds were defined at −1.0, −2.0 and −2.5 standard deviations.

Statistical significance was set at *p* < 0.05 (two‐tailed). All analyses were conducted using JAMOVI software (version 2.3.22 for Windows).

## 3 Results

**FIGURE 1:**
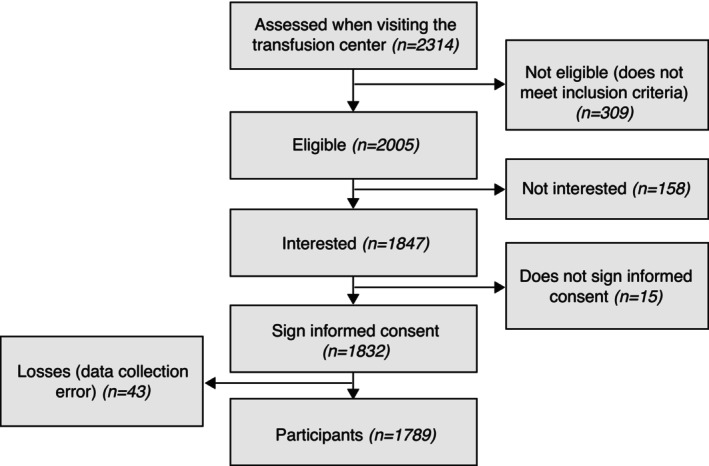
Flow diagram of the participant recruitment process. A total of 2314 individuals were assessed at transfusion centres; 309 were excluded for not meeting inclusion criteria. Reasons for ineligibility included failure to meet eligibility criteria (self‐reported healthy status, absence of physician‐diagnosed chronic disease or regular medication use, and BMI 18.5–35 kg/m^2^), as well as acute or recent illness within the previous month, professional athletic activity, unintentional weight loss > 5% in the previous 3 months, pregnancy or breastfeeding, and the presence of prostheses or other factors likely to interfere with bioelectrical impedance measurements. Of the 2005 eligible individuals, 158 declined participation and 15 did not sign informed consent. After excluding 43 cases due to data collection errors, 1789 participants were included in the final analysis.

A total of 1789 apparently healthy blood donors were included (Figure 1). Baseline sociodemographic characteristics and lifestyle habits are summarised in Table 1. Body composition parameters assessed by anthropometry, BIA, HGS and NU are presented in Table 2.

**TABLE 1:** Baseline characteristics of the study population.

	Overall (*n* = 1789)	Women (*n* = 842)	Men (*n* = 947)	*p*
Age, years, mean ± SD (range)	41.5 ± 14.9 (18–86)	40.6 ± 15.1 (18–86)	42.3 ± 14.7 (18–83)	0.015
Age groups, *n* (%)				0.002
18–30	544 (30.4%)	282 (33.6%)	261 (27.5%)	
31–45	457 (25.6%)	216 (25.7%)	242 (25.5%)	
46–60	584 (32.7%)	239 (28.5%)	346 (36.5%)	
>60	204 (11.3%)	102 (12.2%)	100 (10.5%)	
Educational level, *n* (%)				<0.001
No formal education	15 (0.8%)	6 (0.7%)	9 (0.9%)	
Primary education	133 (7.4%)	49 (5.8%)	84 (8.8%)	
Secondary education	590 (33%)	240 (28.6%)	350 (36.8%)	
Higher education	1051 (58.7%)	544 (64.8%)	507 (53.4%)	
Employment status, *n* (%)				0.018
Unemployed	366 (20.5%)	195 (23.3%)	171 (18%)	
Employed	1315 (73.8%)	596 (71.3%)	719 (75.8%)	
Retired	103 (5.8%)	45 (5.4%)	58 (6.1%)	
Marital status, *n* (%)				0.198
Single	906 (50.7%)	443 (52.8%)	463 (48.8%)	
Married	719 (40.2%)	316 (37.7%)	403 (42.5%)	
Divorced	144 (8.1%)	70 (8.3%)	74 (7.8%)	
Widow/er	18 (1%)	10 (1.2%)	8 (0.8%)	
Smoking status, *n* (%*)*				0.475
Never	1169 (65.4%)	549 (65.4%)	620 (65.4%)	
Former	378 (21.2%)	170 (20.3%)	208 (21.9%)	
Current	240 (13.4%)	120 (14.3%)	120 (12.7%)	
Pack‐year, mean ± SD (range)^a^	3.5 ± 9.3 (0–112)	2.9 ± 8 (0–80)	4 ± 10.2 (0–112)	0.009
Alcohol consumption status, *n* (%)^b^				<0.001
Never	470 (26.3%)	245 (29.2%)	225 (23.7%)	
Former	18 (1%)	4 (0.5%)	14 (1.5%)	
Occasional	1131 (63.2%)	536 (63.9%)	595 (62.6%)	
Regular	170 (9.5%)	54 (6.4%)	116 (12.2%)	
Weekly alcohol units, mean ± SD (range)	3.6 ± 3.9 (0–42)	3 ± 3.1 (0–21)	4.1 ± 4.4 (0–42)	<0.001
Physical activity (IPAQ), MET‐min/week, mean ± SD (range)	2448.8 ± 2102.4 (0–19 860)	1991.4 ± 1540.5 (0–13 491)	2878.6 ± 2442.7 (0–19 860)	<0.001
Physical activity level (IPAQ), *n* (%)				<0.001
Low	127 (8.6%)	73 (10.2%)	54 (7.1%)	
Moderate	999 (67.8%)	534 (74.8%)	465 (61.2%)	
High	348 (23.6%)	107 (15%)	241 (31.7%)	

*Note:* Data are presented as mean ± standard deviation and range (min–max) for continuous variables, and as number (percentage) for categorical variables.

^a^ Pack‐year consumption was calculated cumulatively for both current and former smokers.

^b^ Weekly alcohol units were calculated only for current alcohol consumers, including both occasional and regular users.

**TABLE 2:** Body composition assessed by HSG, BIA and NU.

	Overall (*n* = 1789)		Women (*n* = 842)		Men (*n* = 947)		*p*
	Median (IQR)	Range	Median (IQR)	Range	Median (IQR)	Range	
BMI, kg/m^2^	25.03 (22.86–27.84)	18.51–35.00	24.00 (21.66–26‐42)	18.51–34.94	26.13 (24.06–28.84)	18.70–35.00	<0.001
HGS_med, kg	35.40 (27.90–47.00)	10.00–75.00	27.70 (24.00–30–80)	10.00–55.33	46.00 (39.60–52.00)	14.00–75.00	<0.001
BIA_BCM, kg	29.50 (23.50–35.70)	15.80–50.00	23.30 (21.70–25.20)	15.80–46.90	35.30 (32.40–38.30)	19.90–50.00	<0.001
BIA_ASMM, kg	21.60 (16.90–25.50)	12.00–37.40	16.80 (15.51–18.20)	12.00–30.70	25.30 (23.40–27.30)	15.90–37.40	<0.001
BIA_FM, kg	19.10 (14.90–27.70)	1.90–50.10	19.40 (15.50–24.90)	1.90–49.70	18.70 (14.40–24.50)	3.90–50.10	0.009
T‐ T‐SAT, cm	1.70 (1.15–2.27)	0.00–5.40	1.77 (1.16–2.39)	0.12–5.40	1.65 (1.13–2.22)	0.01–5.03	<0.001
S‐SAT, cm	0.79 (0.53–1.10)	0.00–3.54	0.86 (0.56–1.20)	0.00–3.54	0.76 (0.52–1.03)	0.01–3.28	<0.001
VAT, cm	0.62 (0.43–0.90)	0.08–2.40	0.56 (0.38–0.78)	0.09–2.34	0.71 (0.48–0.98)	0.08–2.40	<0.001
L‐SAT, cm	0.96 (0.63–1.35)	0.00–3.40	1.33 (1.07–1.65)	0.14–3.40	0.67 (0.50–0.90)	0.01–2.66	<0.001
RF‐CSA, cm2	5.65 (4.49–7.06)	2.24–11.90	4.60 (3.83–5.58)	2.24–9.62	6.77 (5.55–7.91)	2.60–11.90	<0.001
RF‐CIR, cm	10.08 (9.02–11.07)	5.61–14.54	9.16 (8.30–9.97)	5.61–13.00	10.87 (10.06–11.59)	7.50–14.54	<0.001
RF‐Xaxis, cm	3.87 (3.44–4.22)	2.18–5.89	3.52 (3.19–3.90)	2.18–5.89	4.12 (3.81–4.37)	2.38–5.83	<0.001
RF‐Yaxis, cm	1.72 (1.47–2.02)	0.69–3.14	1.56 (1.33–1.80)	0.69–2.84	1.91 (1.65–2.17)	0.83–3.14	<0.001
RF‐Yaxis_Contrac, cm	2.21 (1.89–2.58)	0.80–3.83	1.97 (1.71–2.24)	0.80–3.65	2.47 (2.15–2.78)	0.88–3.83	<0.001
Contrac_capacity, cm	0.46 (0.28–0.66)	0.00–2.19	0.39 (0.25–0.56)	0.00–2.19	0.53 (0.34–0.73)	0.01–1.64	<0.001

*Note:* Data are presented as median (interquartile range) and range (min–max).

Abbreviations: BIA‐ASMM, appendicular skeletal muscle mass; BIA‐BCM, body cell mass; BIA‐FM, fat mass; BMI, body mass index; Contrac_capacity, contraction capacity, difference between relaxed and contracted RF‐Y axis measurements; HGS, handgrip strength (mean of three measurements); L‐SAT, superficial adipose tissue over the quadriceps; RF‐CIR, rectus femoris muscle circumference; RF‐CSA, rectus femoris cross‐sectional area; RF‐X axis, transverse axis of the rectus femoris; RF‐Y axis, anteroposterior axis of the rectus femoris at rest; RF‐Y axis_Contrac, anteroposterior axis of the rectus femoris during contraction; S‐SAT, superficial abdominal subcutaneous adipose tissue; T‐SAT, total abdominal subcutaneous adipose tissue; VAT, preperitoneal adipose tissue.

### 3.1 BMI‐Related Differences in NU

Participants with a BMI > 25 kg/m^2^ did not show significant differences in ultrasound‐derived rectus femoris (RF) measurements compared with normal‐weight participants. In contrast, normal‐weight participants exhibited significantly lower abdominal adipose tissue measurements than those with overweight or obesity, consistently in both women and men (all *p* < 0.001; Supplementary Appendix 1A). These differences in abdominal adipose tissue measurements between normal‐weight participants and the higher BMI categories remained statistically significant after indexation to both height squared and body weight.

After indexation of nutritional ultrasound measurements to height squared, rectus femoris measurements in men—except for the X‐axis—showed significant differences between the normal‐weight and obesity groups (all *p* < 0.05). In women, height‐indexed rectus femoris measurements—except for the Y‐axis during contraction—were significantly different between the normal‐weight and overweight groups (all *p* < 0.05; Supplementary Appendix 1B).

When rectus femoris measurements were indexed to body weight, significantly lower values were observed in participants with a BMI > 25 kg/m^2^ compared with normal‐weight participants, in both sexes (all *p* < 0.001; Supplementary Appendix 1C).

### 3.2 Correlations Between NU, BIA, HGS and Anthropometry

**FIGURE 2:**
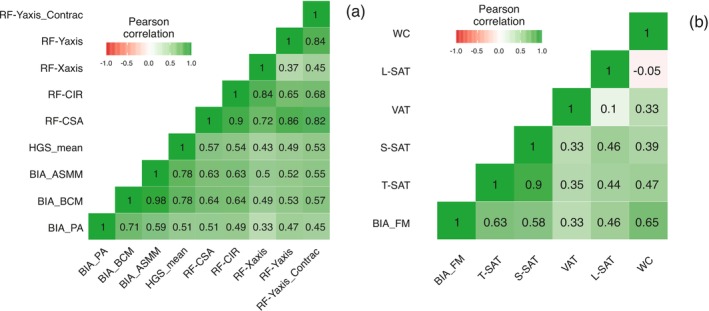
Heatmap of correlations between muscle mass and adipose tissue parameters assessed by NU, BIA, HGS and anthropometry (*n* = 1789). Correlation matrices of morphofunctional parameters. (A) Pearson correlations between bioelectrical impedance parameters (phase angle, body cell mass, appendicular skeletal muscle mass), handgrip strength and rectus femoris ultrasound measurements (cross‐sectional area, circumference, X‐ and Y‐axis, and contracted Y‐axis), showing predominantly moderate to strong positive associations. (B) Pearson correlations between BIA‐derived fat mass and abdominal and thigh adipose tissue ultrasound measurements (total, superficial, visceral and leg subcutaneous adipose tissue) and waist circumference, demonstrating moderate positive correlations overall. Correlation coefficients (*r*) are displayed within each cell. All *p* < 0.001, except for the correlation between L‐SAT and waist circumference, which was weaker (*p* = 0.049). WC, waist circumference; HGS_mean, mean handgrip strength; BIA‐PA, phase angle; BIA‐BCM, body cell mass; BIA_ASMM, appendicular skeletal muscle mass; BIA‐FM, fat mass assessed by bioelectrical impedance analysis; T‐SAT, total abdominal subcutaneous adipose tissue; S‐SAT, superficial abdominal subcutaneous adipose tissue; VAT, preperitoneal adipose tissue assessed by ultrasound; L‐SAT, leg subcutaneous adipose tissue; RF‐CSA, rectus femoris cross‐sectional area; RF‐CIR, rectus femoris circumference; RF‐X axis, transverse axis of the rectus femoris; RF‐Y axis, anteroposterior axis of the rectus femoris at rest; RF‐Y axis (contraction), anteroposterior axis of the rectus femoris during contraction.

Ultrasound‐derived RF measurements showed moderate to strong correlations with BIA‐derived muscle parameters and HGS (all *p* < 0.001) (Figure 2A). RF‐CSA and RF‐CIR showed the strongest correlations with BIA‐derived BCM and ASMM (RF‐CSA: *r* = 0.64 and *r* = 0.63; RF‐CIR: *r* = 0.64 and *r* = 0.63, respectively). Correlations were also observed between BIA‐derived BCM and ASMM and the RF‐Y axis at rest (*r* = 0.53 and *r* = 0.52) and during contraction (*r* = 0.57 and *r* = 0.55). The RF‐X axis showed slightly weaker correlations with BIA‐derived BCM and ASMM (*r* = 0.49 and *r* = 0.50). Phase angle correlated with RF‐CSA, RF‐CIR, RF‐X axis, RF‐Y axis and contracted RF‐Y axis with coefficients of *r* = 0.51, 0.49, 0.33, 0.47 and 0.45, respectively. HGS correlated with RF‐CSA, RF‐CIR, RF‐X axis, RF‐Y axis and contracted RF‐Y axis with coefficients of *r* = 0.57, 0.54, 0.43, 0.49 and 0.53, respectively. Regarding adipose tissue parameters, total abdominal subcutaneous adipose tissue (T‐SAT) showed a moderate correlation with BIA‐derived fat mass (*r* = 0.63) and waist circumference (*r* = 0.47). Superficial abdominal subcutaneous adipose tissue (S‐SAT) correlated with BIA‐derived fat mass (*r* = 0.58) and waist circumference (*r* = 0.39). Preperitoneal adipose tissue (VAT) showed weaker correlations with BIA‐derived fat mass and waist circumference (both *r* = 0.33). Leg subcutaneous adipose tissue (L‐SAT) correlated with BIA‐derived fat mass (*r* = 0.46), but not with waist circumference (*r* = −0.05). (Figure 2B; all *p* < 0.001, except for the correlation between L‐SAT and waist circumference, which was weaker [*p* = 0.049]).

### 3.3 Physical Activity Level (IPAQ) and NU

**FIGURE 3:**
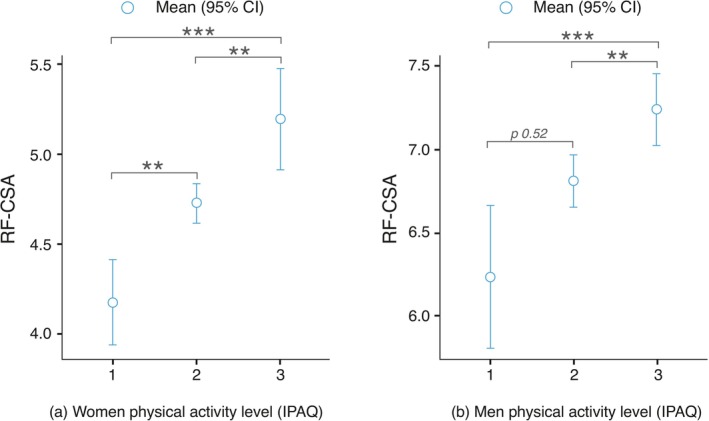
Differences in rectus femoris cross‐sectional area across physical activity levels (IPAQ). Rectus femoris cross‐sectional area according to physical activity level. Mean rectus femoris cross‐sectional area (RF‐CSA) with 95% confidence intervals across IPAQ physical activity groups. (a) Women; (b) men. A progressive increase in RF‐CSA is observed across higher physical activity categories. RF‐CSA, rectus femoris cross‐sectional area; physical activity level (IPAQ) according to self‐reported activity level (1 sedentary, *n* 73 women and 54 men, 2 moderately active, *n* 534 women and 465 men, 3 highly active, *n* 107 women and 241 men). *p < 0.05, **p < 0.01, ***p < 0.001.

Association between self‐reported physical activity levels, assessed using the IPAQ, and RF cross‐sectional area was also examined by sex (Figure 3). In women, RF‐CSA increased progressively across physical activity categories, with significant differences between low and moderate activity (**p < 0.01), low and high activity (***p < 0.001), and moderate and high activity (**p < 0.01). In men, RF‐CSA was significantly higher in the high activity group than in both the low (***p < 0.001) and moderate activity groups (**p < 0.01), whereas no significant difference was observed between low and moderate activity (*p* = 0.52).

### 3.4 Proposed T‐Scores for Nutritional Ultrasound Parameters

#### 3.4.1 NU Assessment of the Rectus Femoris Muscle

All rectus femoris muscle ultrasound parameters met the assumption of normality, with Kolmogorov–Smirnov D statistics ranging from 0.025 to 0.063 and non‐significant *p* values (*p* > 0.05; Supplementary Appendix 2).

No significant differences were observed in any rectus femoris ultrasound measurements between the 18–30 and 30–45 age groups in either women or men (all *p* > 0.05). In contrast, both younger age groups differed significantly from participants aged 45–60 years and those aged over 60 years.

For muscle cross‐sectional area, muscle circumference, anteroposterior (Y) axis and contracted Y axis, significant differences were observed between participants aged 18–45 years and both older age groups (45–60 and > 60 years), with *p* < 0.001 in women and men. Differences in the transverse (X) axis between the 18–45 and 45–60 age groups were smaller but remained statistically significant in both sexes (*p* < 0.05).

Accordingly, participants aged 18–45 years were combined into a single reference group for T‐score derivation (*n* = 1001, stratified by sex, Table 3). Cut‐off points corresponding to −1, −2 and −2.5 standard deviations for muscle ultrasound variables are presented in Table 3. The proportion of individuals below each threshold by age group and sex is reported in Supplementary Appendix 3.

**TABLE 3:** Mean and standard deviation of NU‐derived RF parameters in participants aged 18–45 years (*n* = 1001).

	*Women*					*Men*				
	Mean	SD	−1 SD	−2 SD	−2.5 SD	Mean	SD	−1 SD	−2 SD	−2.5 SD
***RF‐CSA*, cm** ^**2**^	5.13	1.33	3.80	**2.46**	1.80	7.39	1.74	5.70	**3.91**	3.03
***RF‐CIR*, cm**	9.44	1.22	8.22	**7.00**	6.39	11.13	1.23	9.91	**8.68**	8.06
***RF‐Xaxis*, cm**	3.59	0.55	3.04	**2.49**	2.22	4.15	0.48	3.67	**3.20**	2.96
***RF‐Yaxis*, cm**	1.68	0.33	1.36	**1.03**	0.86	2.05	0.369	1.70	**1.31**	1.13
***RF‐Yaxis_Contrac*, cm**	2.10	0.37	1.73	**1.36**	1.17	2.60	0.43	2.17	**1.73**	1.52

*Note:* Mean and standard deviation of NU‐derived RF parameters in participants aged 18–45 years, with corresponding thresholds at −1, −2 and −2.5 SD.

Abbreviations: RF‐CIR, rectus femoris circumference; RF‐CSA, rectus femoris cross‐sectional area; RF‐X axis, transverse axis; RF‐Y axis, anteroposterior axis at rest; RF‐Y axis_Contrac, anteroposterior axis during contraction.

#### 3.4.2 Nutritional Ultrasound of Adipose Tissue

All adipose tissue ultrasound variables failed to meet the assumption of normality in both sexes (T‐SAT, S‐SAT, VAT and L‐SAT, Supplementary Appendix 2). Consequently, T‐scores could not be derived for nutritional ultrasound adipose tissue parameters.

### 3.5 Population‐Based Reference Percentile Values

Age‐ and sex‐specific percentile tables derived from the study population across predefined age groups (18–30, 31–45, 46–60 and > 60 years) are presented as a proposal for their use as population‐based reference values in clinical practice (Tables 4 and 5). In addition, Supplementary Appendixes 4 and 5 include additional percentile tables providing nutritional ultrasound measurements indexed to height squared and to body weight.

**TABLE 4:** Age‐ and sex‐specific percentile values for nutritional ultrasound measurements (women, *n* = 842).

Women 18–30	5th	10th	25th	50th	75th	90th	95th
T‐SAT, cm	0.55	0.70	1.01	1.46	2.04	2.53	2.79
S‐SAT, cm	0.26	0.32	0.46	0.69	1.04	1.31	1.48
VAT, cm	0.21	0.26	0.38	0.56	0.72	0.98	1.14
L‐SAT, cm	0.72	0.81	1.02	1.25	1.56	1.93	2.15
RF‐CSA, cm^2^	3.19	3.66	4.21	5.19	6.09	7.06	7.88
RF‐CIR, cm	7.52	8.11	8.64	9.48	10.30	10.98	11.57
RF‐Xaxis, cm	2.64	2.83	3.26	3.60	4.08	4.23	4.37
RF‐Yaxis, cm	1.16	1.29	1.49	1.70	1.93	2.22	2.34
RF‐Yaxis_Contrac	1.50	1.70	1.90	2.13	2.40	2.63	2.83
**Women 31–45**	***5th***	***10th***	***25th***	***50th***	***75th***	***90th***	***95th***
T‐SAT, cm	0.60	0.79	1.16	1.70	2.23	2.82	3.13
S‐SAT, cm	0.24	0.34	0.57	0.79	1.19	1.48	1.71
VAT, cm	0.20	0.24	0.35	0.54	0.78	1.06	1.35
L‐SAT, cm	0.70	0.86	1.11	1.35	1.61	1.86	2.18
RF‐CSA, cm^2^	3.28	3.52	3.95	4.79	5.73	6.60	7.37
RF‐CIR, cm	7.48	7.83	8.47	9.36	10.12	11.03	11.48
RF‐Xaxis, cm	2.85	2.92	3.19	3.56	3.93	4.27	4.48
RF‐Yaxis, cm	1.16	1.24	1.43	1.61	1.84	1.99	2.08
RF‐Yaxis_Contrac	1.50	1.60	1.79	2.00	2.26	2.50	2.60
**Women 46–60**	***5th***	***10th***	***25th***	***50th***	***75th***	***90th***	***95th***
T‐SAT, cm	0.70	0.88	1.30	2.04	2.66	3.40	3.74
S‐SAT, cm	0.34	0.44	0.65	0.98	1.29	1.75	2.07
VAT, cm	0.18	0.26	0.39	0.57	0.81	1.09	1.35
L‐SAT, cm	0.80	0.86	1.08	1.34	1.70	2.17	2.38
RF‐CSA, cm^2^	2.80	3.01	3.67	4.33	5.12	5.85	6.22
RF‐CIR, cm	7.09	7.51	8.13	9.03	9.70	10.35	10.50
RF‐Xaxis, cm	2.72	2.91	3.15	3.53	3.83	4.12	4.20
RF‐Yaxis, cm	1.02	1.10	1.24	1.46	1.63	1.89	2.05
RF‐Yaxis_Contrac	1.29	1.43	1.62	1.84	2.10	2.31	2.50
**Women > 60**	***5th***	***10th***	***25th***	***50th***	***75th***	***90th***	***95th***
T‐SAT, cm	1.11	1.41	1.75	2.18	2.91	3.44	4.11
S‐SAT, cm	0.44	0.56	0.76	1.03	1.49	1.77	2.04
VAT, cm	0.27	0.32	0.47	0.65	0.86	1.10	1.37
L‐SAT, cm	0.74	0.94	1.12	1.42	1.84	2.10	2.20
RF‐CSA, cm^2^	2.51	2.84	3.27	3.76	4.28	4.99	5.51
RF‐CIR, cm	6.25	6.87	7.90	8.40	9.00	9.68	10.29
RF‐Xaxis, cm	2.63	2.86	3.08	3.32	3.71	3.97	4.11
RF‐Yaxis, cm	0.91	0.98	1.10	1.27	1.52	1.66	1.83
RF‐Yaxis_Contrac	1.19	1.25	1.42	1.63	1.93	2.14	2.39

*Note:* Sample sizes for each age group were as follows: 18–30 years (*n* = 282), 31–45 years (*n* = 216), 46–60 years (*n* = 239), > 60 years (*n* = 102).

Abbreviations: L‐SAT, leg subcutaneous adipose tissue; RF‐CIR, rectus femoris circumference; RF‐CSA, rectus femoris cross‐sectional area; RF‐X axis, transverse axis of the rectus femoris; RF‐Y axis, anteroposterior axis of the rectus femoris at rest; RF‐Y axis (contraction), anteroposterior axis of the rectus femoris during contraction; S‐SAT, superficial abdominal subcutaneous adipose tissue; T‐SAT, total abdominal subcutaneous adipose tissue; VAT, preperitoneal adipose tissue assessed by ultrasound.

**TABLE 5:** Age‐ and sex‐specific percentile values for nutritional ultrasound measurements (men, *n* = 947).

Men 18–30	5th	10th	25th	50th	75th	90th	95th
T‐SAT, cm	0.29	0.45	0.81	1.27	1.90	2.50	3.03
S‐SAT, cm	0.15	0.22	0.39	0.65	0.92	1.22	1.35
VAT, cm	0.24	0.31	0.46	0.67	0.91	1.21	1.43
L‐SAT, cm	0.28	0.33	0.46	0.66	0.96	1.26	1.56
RF‐CSA, cm^2^	4.80	5.22	6.15	7.37	8.72	9.90	10.57
RF‐CIR, cm	9.24	9.65	10.31	11.16	11.92	12.66	13.11
RF‐Xaxis, cm	3.43	3.62	3.84	4.17	4.40	4.76	5.05
RF‐Yaxis, cm	1.51	1.60	1.83	2.07	2.32	2.57	2.71
RF‐Yaxis_Contrac	1.87	2.07	2.28	2.62	2.88	3.16	3.29
**Men 31–45**	***5th***	***10th***	***25th***	***50th***	***75th***	***90th***	***95th***
T‐SAT, cm	0.52	0.68	1.12	1.70	2.11	2.68	2.88
S‐SAT, cm	0.23	0.32	0.49	0.77	1.04	1.32	1.48
VAT, cm	0.26	0.33	0.47	0.67	0.97	1.22	1.56
L‐SAT, cm	0.30	0.37	0.50	0.68	0.93	1.18	1.32
RF‐CSA, cm^2^	4.60	5.06	6.29	7.23	8.44	9.71	10.46
RF‐CIR, cm	9.07	9.58	10.36	11.12	11.90	12.68	13.30
RF‐Xaxis, cm	3.37	3.54	3.82	4.12	4.39	4.60	4.97
RF‐Yaxis, cm	1.42	1.55	1.77	2.00	2.29	2.51	2.62
RF‐Yaxis_Contrac	1.91	2.04	2.27	2.60	2.90	3.16	3.32
**Men 46–60**	***5th***	***10th***	***25th***	***50th***	***75th***	***90th***	***95th***
T‐SAT, cm	0.86	1.07	1.39	1.80	2.34	2.84	3.10
S‐SAT, cm	0.40	0.49	0.61	0.83	1.06	1.30	1.48
VAT, cm	0.29	0.34	0.53	0.77	1.04	1.33	1.42
L‐SAT, cm	0.30	0.36	0.53	0.68	0.86	1.10	1.36
RF‐CSA, cm^2^	4.29	4.71	5.38	6.34	7.21	8.17	9.08
RF‐CIR, cm	8.82	9.29	10.03	10.72	11.24	11.95	12.33
RF‐Xaxis, cm	3.34	3.46	3.81	4.11	4.34	4.51	4.68
RF‐Yaxis, cm	1.27	1.38	1.56	1.79	2.01	2.24	2.41
RF‐Yaxis_Contrac	1.71	1.82	2.07	2.37	2.64	2.93	3.11
**Men >60**	***5th***	***10th***	***25th***	***50th***	***75th***	***90th***	***95th***
T‐SAT, cm	0.86	0.94	1.22	1.64	2.29	2.79	2.94
S‐SAT, cm	0.35	0.40	0.53	0.71	1.03	1.36	1.45
VAT, cm	0.27	0.32	0.43	0.63	0.84	1.12	1.41
L‐SAT, cm	0.30	0.39	0.47	0.63	0.77	1.01	1.30
RF‐CSA, cm^2^	3.49	4.09	4.86	5.62	6.67	7.72	8.16
RF‐CIR, cm	8.60	9.01	9.47	10.15	11.05	11.50	12.09
RF‐Xaxis, cm	3.03	3.34	3.67	4.01	4.24	4.52	4.74
RF‐Yaxis, cm	1.10	1.19	1.42	1.67	1.89	2.08	2.16
RF‐Yaxis_Contrac	1.42	1.58	1.88	2.21	2.49	2.70	2.83

*Note:* Sample sizes for each age group were as follows: 18–30 years (*n* = 261), 31–45 years (*n* = 242), 46–60 years (*n* = 364), >60 years (*n* = 100).

Abbreviations: L‐SAT, leg subcutaneous adipose tissue; RF‐CIR, rectus femoris circumference; RF‐CSA, rectus femoris cross‐sectional area; RF‐X axis, transverse axis of the rectus femoris; RF‐Y axis, anteroposterior axis of the rectus femoris at rest; RF‐Y axis (contraction), anteroposterior axis of the rectus femoris during contraction; S‐SAT, superficial abdominal subcutaneous adipose tissue; T‐SAT, total abdominal subcutaneous adipose tissue; VAT, preperitoneal adipose tissue assessed by ultrasound.

## 4 Discussion

This study presents, for the first time, reference values for nutritional ultrasound measurements in an apparently healthy adult population across adulthood (18–80 years) in Spain. These include sex‐ and age‐specific reference values for quadriceps muscle obtained using a standardised ultrasound technique at the distal third of the rectus femoris, together with proposed cut‐off points for defining low muscle mass in the context of malnutrition and sarcopenia. In addition, reference values for superficial subcutaneous and preperitoneal abdominal adipose tissue measured at the abdominal midpoint between the umbilicus and the xiphoid process are reported.

The establishment of normative nutritional ultrasound values is essential for defining sex‐specific cut‐off points for conditions such as sarcopenia, sarcopenic obesity and disease‐related malnutrition, as well as for predicting clinically relevant outcomes including morbidity and mortality. Moreover, the present study explores the associations between ultrasound‐derived muscle and adipose tissue measurements and other body composition techniques, muscle strength, physical activity levels and BMI categories. BMI, despite its well‐recognised limitations in reflecting body composition [29, 30], remains widely used in clinical practice and epidemiological research, which supports its inclusion as a pragmatic benchmark. Importantly, these reference values are derived from an apparently healthy population, in contrast to most previously published studies, which have largely focused on hospitalised or outpatient populations with established disease [11, 12, 14, 18, 31, 32, 33]. However, the proposed reference values should be considered as population‐based estimates derived from this cohort, rather than definitive universal standards.

As expected, all assessed body composition parameters differed significantly between women and men, underscoring the necessity of applying sex‐specific reference values and cut‐off points in body composition research and clinical practice.

In our study, quadriceps nutritional ultrasound measurements demonstrated appropriate convergent validity, showing moderate to strong correlations with other muscle mass assessment techniques, including BIA‐derived muscle parameters, and muscle function as assessed by handgrip strength. These findings support the validity of nutritional ultrasound as a reliable method for the assessment of skeletal muscle quantity and function. BIA‐derived variables were included as clinically relevant comparators, reflecting a widely used and accessible method for body composition assessment. This approach allows the findings to be interpreted within the context of current clinical practice, while acknowledging that BIA does not represent a gold‐standard technique for the direct assessment of muscle mass. BIA‐derived variables were selected based on their physiological relevance to complementary dimensions of muscle status. Specifically, body cell mass (BCM), appendicular skeletal muscle mass (ASMM) and phase angle (PA) are widely used in clinical practice and have demonstrated prognostic and diagnostic value, supporting their use as clinically meaningful comparators to explore convergent validity [2, 30, 34, 35].

Regarding adipose tissue, L‐SAT, S‐SAT and T‐SAT measured by nutritional ultrasound showed moderate correlations with BIA‐derived fat mass. In contrast, preperitoneal adipose tissue exhibited weaker correlations with BIA. This finding may be explained by the right‐skewed population distribution of preperitoneal fat and its closer association with low‐grade inflammation and metabolic risk factors, such as obesity and insulin resistance, rather than with simple anthropometric measures.

Population‐based percentile distributions stratified by age and sex were generated for all nutritional ultrasound parameters. In addition, T‐scores for quadriceps muscle measurements were derived using a young reference population aged 18–45 years and are intended to be applied across the adult age range to identify low muscle mass, following an approach analogous to that used in densitometry. This approach allows for the identification of clinically relevant low muscle mass independent of age‐related physiological decline. When applying these T‐scores to participants aged 45–60 years and over 60 years, a threshold of −1 SD classified an excessively high proportion of individuals as having low muscle mass (ranging from 13.7% to 46.5%) despite the apparently healthy nature of the cohort. Conversely, a −2.5 SD threshold appeared overly restrictive, identifying only 0.2%–5.6% of individuals aged over 45 years. A threshold of −2 SD identified between 1.4% and 11.7% of individuals aged over 45 years as having low muscle mass, representing a more balanced and clinically plausible proportion. Accordingly, we propose the use of −2 standard deviations from the mean of the healthy adult population under 45 years of age as a reference cut‐off for defining low muscle mass in rectus femoris ultrasound measurements (Table 3). T‐scores were not calculated for adipose tissue measurements due to the lack of normal distribution of these variables. Alternatively, population percentile tables by age and sex are also provided; in many groups, the 5th percentile is very similar to the selected T‐score threshold of −2 SD. The proposed T‐score thresholds provide a pragmatic framework for identifying low muscle mass; however, their clinical validity and diagnostic performance require further evaluation in independent and disease‐specific populations. In future studies, it will be of interest to compare the suitability of applying different cut‐off points to define low muscle mass.

Nutritional ultrasound measurements also demonstrated discriminative validity, as quadriceps muscle cross‐sectional area was positively associated with self‐reported physical activity levels assessed using the IPAQ. Participants with higher physical activity levels consistently exhibited greater rectus femoris muscle area, supporting the functional relevance of the ultrasound‐derived measurements.

Although normative muscle ultrasound data remain scarce, external comparisons support the robustness of our findings. The cut‐off points proposed in the DRECO study (García‐Almeida et al., 2023a), conducted in hospitalised patients at nutritional risk, are remarkably similar to those observed in our apparently healthy population, despite substantial differences in clinical context. Likewise, ultrasound thresholds reported in the VALOR project [14], associated with malnutrition severity in patients with head and neck cancer, converge with the lower percentiles of our reference population.

Other studies further reinforce the convergent validity and prognostic value of nutritional ultrasound. In cystic fibrosis, rectus femoris ultrasound parameters have shown strong correlations with DXA, BIA, CT and handgrip strength [13], supporting their utility in chronic disease settings. In oncological populations, reductions in the anteroposterior axis of the rectus femoris and decreases in BIA‐derived parameters have been associated with worse nutritional status, greater malnutrition severity according to GLIM criteria and increased mortality, highlighting the prognostic relevance of muscle ultrasound [10, 14, 36, 37]. Our approach is conceptually aligned with population‐based studies that have established normative reference values for muscle‐related parameters using DXA, CT and handgrip strength [27, 38, 39, 40, 41]. Although methodological differences preclude direct comparison, these studies share a common framework based on age‐ and sex‐specific reference distributions, supporting the rationale of the present work.

In individuals with obesity attending endocrinology and nutrition clinics, with a mean BMI of approximately 35 kg/m^2^, studies using the same ultrasound technique have reported correlations between rectus femoris cross‐sectional area, fat‐free mass, body cell mass estimated by BIA and handgrip strength that are highly consistent with those observed in our cohort [3]. Given that individuals with obesity often present higher absolute muscle mass according to BIA or DXA but similar rectus femoris dimensions, we propose the use of muscle circumference indexed to body weight or height, with values below the 5th percentile, as a potential criterion for defining sarcopenic obesity. Thus, the prevalence of sarcopenic obesity will not be underestimated, as can occur when fat‐free mass or appendicular skeletal muscle mass is indexed to height squared, nor overestimated if these indices are instead normalised to body weight [8, 30].

When comparing our findings with studies assessing the rectus femoris at the midpoint between the anterior superior iliac spine and the patella, it should be noted that the muscle is typically larger at this site. In healthy individuals it may even exceed the linear probe field of view, compromising complete and reproducible measurements. Therefore, we performed assessments at the lower third of the thigh, a more homogeneous region that facilitates consistent image acquisition. Previous ultrasound studies in the field of sarcopenia, such as those conducted in older populations, assessed quadriceps muscle parameters at the mid‐thigh level. These studies were performed exclusively in elderly subjects and used different anatomical landmarks compared with the present study, which limits the direct comparability of absolute muscle measurements [16].

Future research should aim to establish the minimal clinically important difference for nutritional ultrasound measurements to allow meaningful interpretation of longitudinal changes. In addition, the progressive integration of artificial intelligence–based algorithms may further improve precision, reproducibility and diagnostic performance, expanding the applicability of nutritional ultrasound in both clinical practice and research [42].

### 4.1 Strengths

This study has several strengths. First, it includes a large sample size and was conducted across multiple centres, enhancing its robustness and potential representativeness of the adult Spanish population. The study population comprised apparently healthy blood donors, allowing the generation of reference values derived from a non‐clinical cohort, in contrast to most previous studies performed in hospitalised or outpatient populations.

The majority of participants were of Caucasian ethnicity, providing a homogeneous reference population and reducing ethnic variability in body composition measurements. In addition, nutritional ultrasound assessments were performed using a standardised technique described by García‐Almeida et al. [5], with measurements obtained at the distal third of the thigh. This anatomical location is easily identifiable and less susceptible to anatomical variation, favouring clinical applicability, inter‐observer agreement, and reproducibility. All investigators involved in the study were trained in this methodology, facilitating comparability with current and future clinical studies using the same standardised protocol.

### 4.2 Limitations

Several limitations should be acknowledged. First, this was not a population‐based study selected directly from census data, which may limit full population representativeness. In this regard, the proportion of participants with higher education was relatively high, and self‐reported physical activity levels may exceed those observed in the general population. These factors suggest a potential selection bias towards a healthier and more health‐conscious cohort. In addition, the number of participants aged over 65 years was lower than in younger age groups, reflecting the reduced proportion of blood donors in this age range. Importantly, the proposed reference values should be interpreted in the context of a predominantly Caucasian population, which may limit their generalisability to populations with different ethnic backgrounds.

Participants with BMI values between 25 and 35 kg/m^2^ were not excluded, which may be questioned when defining an ostensibly healthy population. However, given the high prevalence of overweight and obesity in the general population [43], excluding these individuals would have reduced representativeness. Moreover, analyses stratified by BMI and indexed to body weight were performed to account for this factor, particularly in the assessment of sarcopenia.

A further limitation of this study relates to the use of BIA as a comparator method. Although BIA is recognised as an accepted method for the assessment of muscle mass within the Global Leadership Initiative on Malnutrition (GLIM) criteria [1], it remains an indirect technique that estimates body composition based on electrical properties and does not directly measure muscle mass. Nevertheless, BIA was selected due to its feasibility and applicability within the context of this study, which included a large cohort of apparently healthy individuals recruited in a real‐world setting. Importantly, although the exclusion criteria reduced the likelihood of key GLIM phenotypic and etiologic components, GLIM criteria were not formally applied in this study, and participants were not excluded based on low muscle mass as assessed by BIA or handgrip strength.

Additionally, ultrasound measurements were limited to a single anatomical location, and assessment of additional muscle or adipose tissue sites could have provided complementary information. Participants were not required to remain supine for a standardised resting period before ultrasound and BIA assessment, reflecting real‐world clinical conditions. As a result, fluid shifts related to body position may have influenced some measurements. Furthermore, muscle quality parameters were not evaluated, and only quantitative muscle measurements were assessed, despite the known prognostic relevance of muscle composition. Finally, no biochemical measurements were available to explore potential associations between preperitoneal adipose tissue and inflammatory or metabolic markers, such as glycaemia or insulin resistance.

## 5 Conclusions

This study provides, for the first time, age‐ and sex‐specific reference values for nutritional ultrasound measurements in an apparently healthy adult Spanish population, together with proposed cut‐off points for the definition of low muscle mass.

These findings establish a foundation for future research aimed at validating the proposed cut‐off points in other populations and assessing their diagnostic performance and concordance with other body composition techniques for the identification of malnutrition, sarcopenia and sarcopenic obesity, as well as their ability to predict adverse clinical outcomes.

The availability of population‐based reference values represents an important step towards the standardisation of nutritional ultrasound and supports its routine use in clinical practice. Furthermore, these reference data and proposed cut‐off points may facilitate their application in future population‐based and clinical studies.

## Author Contributions

G.O. and J.M.G.‐A. designed research studies. M.G.‐O., G.O., J.M.G.‐A., M.L.‐d.‐l.‐T.‐C. and P.P.G.‐L. supervised the study. Recruitment of participants, data collection and nutritional assessment were performed by J.M.R.‐M., M.G.‐O., M.N.‐R., R.F.‐J., I.V.‐A., S.G.‐R., A.M.‐G., C.N.‐R., J.M.G.‐B., M.P.‐B., N.P.‐P., M.C.R.‐C., A.J.‐S. and A.J.M.‐O. Data curation was performed by C.M.‐T.‐T and M.G.‐O. Statistics analysis was provided by C.M.‐T.‐T., M.G.‐O., G.O. and G.R.‐M. C.M.‐T.‐T. wrote the manuscript. All authors revised the manuscript. All authors have read and agreed to the published version of the manuscript.

## Funding

This study was partially funded by an unrestricted grant from FRESENIUS KABI (Spain). The APC was jointly supported by Fresenius Kabi Spain and the Málaga Biomedical Research Institute (IBIMA–BIONAND Platform). Gemma Rojo‐Martinez belongs to the Nicolás Monardes research program of the Regional Ministry of Health of Andalusia (C‐0060‐2012; Junta de Andalucia, Spain).

## Ethics Statement

The study was conducted in accordance with the Declaration of Helsinki and approved by the Research Ethics Committee of Malaga on 31 August 2023.

## Consent

Informed consent was obtained from all subjects involved in the study.

## Conflicts of Interest

The authors declare no conflicts of interest.

## Supporting information


**Appendix S1:** Differences in nutritional ultrasound (NU) measurements across BMI categories.
**Appendix S2:** Normality test for NU measurements.
**Appendix S3:** % of participants below −1SD, −2SD and −2.5SD towards our T‐score proposal.
**Appendix S4:** Percentiles of NU measurements (cm) between height squared (m2).
**Appendix S5:** Percentile tables of NU measurements (cm) between weight (kg) multiplied by 100.

## Data Availability

The datasets generated and/or analysed during the current study are not publicly available due to ethical and data protection restrictions but are available from the corresponding author on reasonable request, subject to approval by the appropriate ethics committee and applicable institutional and data protection regulations.
