# Supplementary material for: Normative Reference Values and Cut‐Off Points for Nutritional Ultrasound: The VALORES Study
Source: J Cachexia Sarcopenia Muscle. 2026 Aug 19;17(4):e70344. doi: 10.1002/jcsm.70344 (PMC13487697; doi:10.1002/jcsm.70344)
Supplement: Supplementary file 1 [file JCSM-17-e70344-s001.docx]

**SUPPLEMENTARY MATERIAL**

**Supplementary Appendix 1. Differences in nutritional ultrasound (NU) measurements across BMI categories**

A. Differences in absolute NU measurements

| Variable | Men: (Welch) | Men: significant post-hoc (p)* | Women: (Welch) | Women: significant post-hoc (p)* |
| --- | --- | --- | --- | --- |
| **T-SAT** | <0.001 | Normal vs Overweight <0.001; Normal vs Obesity <0.001; Overweight vs Obesity <0.001 | <0.001 | Normal vs Overweight <0.001; Normal vs Obesity <0.001; Overweight vs Obesity <0.001 |
| **S-SAT** | <0.001 | Normal vs Overweight <0.001; Normal vs Obesity <0.001; Overweight vs Obesity <0.001 | <0.001 | Normal vs Overweight <0.001; Normal vs Obesity <0.001; Overweight vs Obesity <0.001 |
| **VAT** | <0.001 | Normal vs Overweight <0.001; Normal vs Obesity <0.001; Overweight vs Obesity <0.001 | <0.001 | Normal vs Overweight <0.001; Normal vs Obesity <0.001; Overweight vs Obesity = 0.012 |
| **L-SAT** | <0.001 | Normal vs Overweight <0.001; Normal vs Obesity <0.001; Overweight vs Obesity <0.001 | <0.001 | Normal vs Overweight <0.001; Normal vs Obesity <0.001; Overweight vs Obesity <0.001 |
| **RF-CSA** | 0.223 | — | 0.049 | — |
| **RF-CIR** | 0.182 | — | 0.100 | — |
| **RF-X axis** | 0.494 | — | 0.068 | — |
| **RF-Y axis** | 0.050 | — | 0.055 | — |
| **RF-Y axis co.** | 0.158 | — | 0.250 | — |

**S.a.1A** Between-group differences were assessed using Welch’s ANOVA. When significant, pairwise comparisons were performed using the Games–Howell post-hoc test. Only significant post-hoc comparisons are displayed.

**Body mass index (BMI) Groups:** Normal BMI 18-25, Overweight BMI 25-30, Obesity BMI 30-35

**T-SAT**, total abdominal subcutaneous adipose tissue; **S-SAT**, superficial abdominal subcutaneous adipose tissue; **VAT**, preperitoneal adipose tissue assessed by ultrasound; **L-SAT**, leg subcutaneous adipose tissue**; RF-CSA**, rectus femoris cross-sectional area; **RF-CIR**, rectus femoris circumference; **RF-X axis**, transverse axis of the rectus femoris; **RF-Y axis**, anteroposterior axis of the rectus femoris at rest; **RF-Y axis (contraction)**, anteroposterior axis of the rectus femoris during contraction

Pairwise mean differences are reported as Normal weight − comparator. For abdominal adipose tissue, mean differences were consistently negative (higher values with increasing BMI): in men, Normal vs Overweight ranged from −0.536 to −0.154, Normal vs Obesity from −0.990 to −0.289, and Overweight vs Obesity from −0.454 to −0.121; in women, the corresponding ranges were −1.030 to −0.206, −1.449 to −0.325, and −0.422 to −0.119, respectively. Rectus femoris measurements showed small, non-directional mean differences, with no consistent trend across BMI groups.

B. Differences in height-indexed NU measurements (per height squared)

| Variable | Men (Welch) | Men: significant post-hoc (p)* | Women (Welch) | Women: significant post-hoc (p)* |
| --- | --- | --- | --- | --- |
| **T-SAT/h²** | <0.001 | Normal–Overweight <0.001; Normal–Obesity <0.001; Overweight–Obesity <0.001 | <0.001 | Normal–Overweight <0.001; Normal–Obesity <0.001; Overweight–Obesity <0.001 |
| **S-SAT/h²** | <0.001 | Normal–Overweight <0.001; Normal–Obesity <0.001; Overweight–Obesity <0.001 | <0.001 | Normal–Overweight <0.001; Normal–Obesity <0.001; Overweight–Obesity <0.001 |
| **VAT/h²** | <0.001 | Normal–Overweight <0.001; Normal–Obesity <0.001; Overweight–Obesity <0.001 | <0.001 | Normal–Overweight <0.001; Normal–Obesity <0.001; Overweight–Obesity 0.006 |
| **L-SAT/h²** | <0.001 | Normal–Overweight <0.001; Normal–Obesity <0.001; Overweight–Obesity <0.001 | <0.001 | Normal–Overweight <0.001; Normal–Obesity <0.001; Overweight–Obesity <0.001 |
| **RFCSA/h²** | 0.025 | Normal–Obesity 0.034 | 0.007 | Normal–Overweight 0.017 |
| **RFCIR/h²** | 0.013 | Normal–Obesity 0.009 | 0.003 | Normal–Overweight 0.003 |
| **RFXaxis/h²** | 0.376 | — | 0.008 | Normal–Overweight 0.005 |
| **RFYaxis/h²** | <0.001 | Normal–Obesity 0.001 | 0.004 | Normal–Obesity 0.008 |
| **RFYaxisco/h²** | 0.008 | Normal–Obesity 0.006 | 0.030 | — |

**S.a.1B** Between-group differences were assessed using Welch’s ANOVA. When significant, pairwise comparisons were performed using the Games–Howell post-hoc test. Only significant post-hoc comparisons are displayed.

**Body mass index (BMI) Groups:** Normal BMI 18-25, Overweight BMI 25-30, Obesity BMI 30-35

**T-SAT**, total abdominal subcutaneous adipose tissue; **S-SAT**, superficial abdominal subcutaneous adipose tissue; **VAT**, preperitoneal adipose tissue assessed by ultrasound; **L-SAT**, leg subcutaneous adipose tissue**; RF-CSA**, rectus femoris cross-sectional area; **RF-CIR**, rectus femoris circumference; **RF-X axis**, transverse axis of the rectus femoris; **RF-Y axis**, anteroposterior axis of the rectus femoris at rest; **RF-Y axis (contraction)**, anteroposterior axis of the rectus femoris during contraction

Pairwise mean differences are reported as Normal weight − comparator. Abdominal adipose tissue differences remained negative across BMI categories (higher values with increasing BMI): in men, Normal vs Overweight ranged from −0.176 to −0.051, Normal vs Obesity from −0.331 to −0.099, and Overweight vs Obesity from −0.156 to −0.044; in women, the corresponding ranges were −0.396 to −0.081, −0.561 to −0.130, and −0.165 to −0.049, respectively. Height-indexed rectus femoris measurements also showed negative mean differences where significant: in men, Normal vs Obesity ranged from −0.1300 to −0.0431; in women, significant Normal vs Overweight differences ranged from −0.1340 to −0.0512, and Normal vs Obesity for RF Y-axis was −0.0481

C. Differences in weight-indexed NU measurements

| Variable | Men: p global (Welch) | Men: significant post-hoc (p)* | Women: p global (Welch) | Women: significant post-hoc (p)* |
| --- | --- | --- | --- | --- |
| **T-SAT/weight** | <0.001 | Normal–Overweight <0.001; Normal–Obesity <0.001 | <0.001 | Normal–Overweight <0.001; Normal–Obesity <0.001 |
| **S-SAT/weight** | <0.001 | Normal–Overweight <0.001; Normal–Obesity <0.001 | <0.001 | Normal–Overweight <0.001; Normal–Obesity <0.001 |
| **VAT/weight** | 0.051 | — | <0.001 | Normal–Overweight <0.001 |
| **L-SAT/weight** | <0.001 | Normal–Overweight 0.047; Normal–Obesity <0.001; Overweight–Obesity 0.009 | 0.002 | Normal–Overweight 0.017; Normal–Obesity 0.015 |
| **RF-CSA/weight** | <0.001 | Normal–Overweight <0.001; Normal–Obesity <0.001; Overweight–Obesity <0.001 | <0.001 | Normal–Overweight <0.001; Normal–Obesity <0.001; Overweight–Obesity <0.001 |
| **RF-CIR/weight** | <0.001 | Normal–Overweight <0.001; Normal–Obesity <0.001; Overweight–Obesity <0.001 | <0.001 | Normal–Overweight <0.001; Normal–Obesity <0.001; Overweight–Obesity <0.001 |
| **RF-X axis/weight** | <0.001 | Normal–Overweight <0.001; Normal–Obesity <0.001; Overweight–Obesity <0.001 | <0.001 | Normal–Overweight <0.001; Normal–Obesity <0.001; Overweight–Obesity <0.001 |
| **RF-Y axis/weight** | <0.001 | Normal–Overweight <0.001; Normal–Obesity <0.001; Overweight–Obesity <0.001 | <0.001 | Normal–Overweight <0.001; Normal–Obesity <0.001; Overweight–Obesity <0.001 |
| **RF-Y axis (contraction)/weight** | <0.001 | Normal–Overweight <0.001; Normal–Obesity <0.001; Overweight–Obesity <0.001 | <0.001 | Normal–Overweight <0.001; Normal–Obesity <0.001; Overweight–Obesity <0.001 |

**S.a.1C** Between-group differences were assessed using Welch’s ANOVA. When significant, pairwise comparisons were performed using the Games–Howell post-hoc test. Only significant post-hoc comparisons are displayed.

**Body mass index (BMI) Groups:** Normal BMI 18-25, Overweight BMI 25-30, Obesity BMI 30-35

**T-SAT**, total abdominal subcutaneous adipose tissue; **S-SAT**, superficial abdominal subcutaneous adipose tissue; **VAT**, preperitoneal adipose tissue assessed by ultrasound; **L-SAT**, leg subcutaneous adipose tissue**; RF-CSA**, rectus femoris cross-sectional area; **RF-CIR**, rectus femoris circumference; **RF-X axis**, transverse axis of the rectus femoris; **RF-Y axis**, anteroposterior axis of the rectus femoris at rest; **RF-Y axis (contraction)**, anteroposterior axis of the rectus femoris during contraction.

Pairwise mean differences are reported as Normal weight − comparator. For abdominal adipose tissue, mean differences were negative where significant (higher values in overweight/obesity): in men, Normal vs Overweight ranged from −0.392 to −0.065 and Normal vs Obesity from −0.546 to −0.167 (Overweight vs Obesity for L-SAT/weight: −0.103); in women, Normal vs Overweight ranged from −1.060 to −0.134 and Normal vs Obesity from −1.033 to −0.210. In contrast, rectus femoris measurements indexed to body weight showed consistently positive mean differences (lower values in overweight/obesity): in men, Normal vs Overweight ranged from +0.315 to +1.94, Normal vs Obesity from +0.607 to +3.81, and Overweight vs Obesity from +0.292 to +1.87; in women, the corresponding ranges were +0.382 to +2.21, +0.651 to +4.43, and +0.270 to +2.22

**Supplementary apendix 2: Normality test for NU measurements**

**S.a.2** Distribution of nutritional ultrasound measurements in adults aged 18–45 years (n = 1,001). Sex-specific histograms (0 = women, 1 = men) illustrating the distribution of rectus femoris cross-sectional area, circumference, transverse and anteroposterior muscle thickness, total subcutaneous adipose tissue, and visceral adipose tissue. Distributions are shown to assess normality assumptions for subsequent parametric analyses.

**CSA**, rectus femoris cross-sectional area; **CIR**, muscle circumference; **X axis**, transverse muscle axis; **Y axis**, anteroposterior muscle axis; **T-SAT**, total abdominal subcutaneous adipose tissue; **VAT**, visceral (preperitoneal) adipose tissue.

*p-values: RF-CSA, p = 0.016; RF-CIR, p = 0.952; RF-X axis, p = 0.579; RF-Y axis, p = 0.173; T-SAT, p = 0.024; VAT, p < 0.001*

**Supplementary appendix 3: % of participants below -1SD, -2SD and -2.5SD towards our T-score proposal**

|  | **45-60 years** | | | **>60 years** | | |
| --- | --- | --- | --- | --- | --- | --- |
|  | **-1DE** | **-2DE** | **-2,5DE** | **-1 DE** | **-2DE** | **-2,5DE** |
| *RF-CSA, cm2* | 29.5% | **1.7%** | 0.2% | 46.5% | **5%** | 0.5% |
| *RF-CIR, cm* | 19.2% | **3.9%** | 0.9% | 31.7% | **5.3%** | 4.2% |
| *RF-Xaxis, cm* | 13.7% | **1.4%** | 1% | 22.3% | **2.5%** | 1.5% |
| *RF-Yaxis, cm* | 32.5% | **4.8%** | 1.5% | 40.1% | **11.4%** | 4.5% |
| *RF-Yaxis_Contrac, cm* | 15.3% | **4.2%** | 1.5% | 36.2% | **11.7%** | 5.6% |

**S.a. 3** Percentage of participants aged 45–60 years and >60 years presenting −1 SD, −2 SD and −2.5 SD relative to the T-score derived from the 18–45-year reference sample in muscle ultrasound.

RF-CSA: rectus femoris cross-sectional area; RF-CIR: rectus femoris circumference; RF-Xaxis: rectus femoris X-axis; RF-Yaxis: rectus femoris Y-axis; RF-Yaxis_Contrac: rectus femoris Y-axis during contraction.

**Supplementary apendix 4: Percentiles of NU measurements (cm) between height squared (m2)**

| **Women 18-30** | ***5th*** | ***10th*** | ***25th*** | ***50th*** | ***75th*** | ***90th*** | ***95th*** |
| --- | --- | --- | --- | --- | --- | --- | --- |
| T-SAT/h2 | 0.21 | 0.25 | 0.37 | 0.53 | 0.75 | 0.94 | 1.07 |
| S-SAT/h2 | 0.09 | 0.12 | 0.17 | 0.26 | 0.38 | 0.48 | 0.57 |
| VAT/h2 | 0.08 | 0.10 | 0.14 | 0.20 | 0.27 | 0.38 | 0.43 |
| L-SAT/h2 | 0.26 | 0.30 | 0.37 | 0.47 | 0.58 | 0.74 | 0.82 |
| RF-CSA/h2 | 1.22 | 1.33 | 1.58 | 1.92 | 2.25 | 2.53 | 2.89 |
| RF-CIR/h2 | 2.74 | 2.89 | 3.16 | 3.51 | 3.81 | 4.11 | 4.27 |
| RF-Xaxis/h2 | 0.96 | 1.05 | 1.17 | 1.35 | 1.49 | 1.59 | 1.69 |
| RF-Yaxis/h2 | 0.44 | 0.47 | 0.55 | 0.63 | 0.71 | 0.80 | 0.90 |
| Yaxis_Contrac | 0.58 | 0.63 | 0.69 | 0.80 | 0.90 | 0.99 | 1.05 |
| **Women 31-45** | ***5th*** | ***10th*** | ***25th*** | ***50th*** | ***75th*** | ***90th*** | ***95th*** |
| T-SAT/h2 | 0.20 | 0.29 | 0.29 | 0.60 | 0.83 | 1.04 | 1.20 |
| S-SAT/h2 | 0.09 | 0.13 | 0.13 | 0.30 | 0.45 | 0.55 | 0.67 |
| VAT/h2 | 0.07 | 0.09 | 0.09 | 0.20 | 0.28 | 0.39 | 0.51 |
| L-SAT/h2 | 0.25 | 0.31 | 0.31 | 0.50 | 0.59 | 0.72 | 0.79 |
| RF-CSA/h2 | 1.18 | 1.32 | 1.32 | 1.75 | 2.08 | 2.47 | 2.79 |
| RF-CIR/h2 | 2.73 | 2.91 | 2.91 | 3.38 | 3.73 | 4.11 | 4.39 |
| RF-Xaxis/h2 | 1.02 | 1.07 | 1.07 | 1.28 | 1.46 | 1.57 | 1.73 |
| RF-Yaxis/h2 | 0.42 | 0.46 | 0.46 | 0.60 | 0.68 | 0.74 | 0.79 |
| Yaxis_Contrac | 0.56 | 0.59 | 0.59 | 0.74 | 0.84 | 0.94 | 0.98 |
| **Women 46-60** | ***5th*** | ***10th*** | ***25th*** | ***50th*** | ***75th*** | ***90th*** | ***95th*** |
| T-SAT/h2 | 0.26 | 0.33 | 0.47 | 0.79 | 1.01 | 1.27 | 1.39 |
| S-SAT/h2 | 0.11 | 0.16 | 0.24 | 0.37 | 0.50 | 0.66 | 0.78 |
| VAT/h2 | 0.07 | 0.10 | 0.15 | 0.21 | 0.30 | 0.40 | 0.51 |
| L-SAT/h2 | 0.27 | 0.32 | 0.40 | 0.51 | 0.64 | 0.82 | 0.88 |
| RF-CSA/h2 | 1.02 | 1.18 | 1.36 | 1.62 | 1.93 | 2.19 | 2.41 |
| RF-CIR/h2 | 2.63 | 2.76 | 3.05 | 3.32 | 3.65 | 3.94 | 4.08 |
| RF-Xaxis/h2 | 1.01 | 1.09 | 1.18 | 1.29 | 1.45 | 1.57 | 1.63 |
| RF-Yaxis/h2 | 0.38 | 0.40 | 0.46 | 0.54 | 0.62 | 0.70 | 0.77 |
| Yaxis_Contrac | 0.48 | 0.54 | 0.60 | 0.70 | 0.80 | 0.89 | 0.92 |
| **Women >60** | ***5th*** | ***10th*** | ***25th*** | ***50th*** | ***75th*** | ***90th*** | ***95th*** |
| T-SAT/h2 | 0.43 | 0.53 | 0.67 | 0.84 | 1.16 | 1.45 | 1.58 |
| S-SAT/h2 | 0.15 | 0.22 | 0.29 | 0.41 | 0.57 | 0.74 | 0.80 |
| VAT/h2 | 0.10 | 0.13 | 0.19 | 0.25 | 0.33 | 0.44 | 0.55 |
| L-SAT/h2 | 0.28 | 0.34 | 0.44 | 0.56 | 0.73 | 0.85 | 0.91 |
| RF-CSA/h2 | 1.056 | 1.11 | 1.28 | 1.46 | 1.64 | 1.97 | 2.10 |
| RF-CIR/h2 | 2.45 | 2.69 | 3.03 | 3.21 | 3.51 | 3.74 | 3.94 |
| RF-Xaxis/h2 | 1.02 | 1.09 | 1.19 | 1.31 | 1.41 | 1.56 | 1.64 |
| RF-Yaxis/h2 | 0.36 | 0.38 | 0.42 | 0.50 | 0.57 | 0.66 | 0.71 |
| Yaxis_Contrac | 0.46 | 0.50 | 0.56 | 0.64 | 0.74 | 0.84 | 0.95 |

| **Men 18-30** | ***5th*** | ***10th*** | ***25th*** | ***50th*** | ***75th*** | ***90th*** | ***95th*** |
| --- | --- | --- | --- | --- | --- | --- | --- |
| T-SAT/h2 | 0.10 | 0.14 | 0.26 | 0.40 | 0.59 | 0.80 | 0.98 |
| S-SAT/h2 | 0.05 | 0.07 | 0.12 | 0.20 | 0.29 | 0.40 | 0.45 |
| VAT/h2 | 0.06 | 0.097 | 0.15 | 0.22 | 0.29 | 0.39 | 0.45 |
| L-SAT/h2 | 0.09 | 0.10 | 0.15 | 0.21 | 0.31 | 0.40 | 0.50 |
| RF-CSA/h2 | 1.50 | 1.63 | 1.96 | 2.32 | 2.68 | 3.12 | 3.41 |
| RF-CIR/h2 | 2.87 | 3.00 | 3.22 | 3.51 | 3.86 | 4.18 | 4.35 |
| RF-Xaxis/h2 | 1.08 | 1.13 | 1.21 | 1.32 | 1.43 | 1.52 | 1.60 |
| RF-Yaxis/h2 | 0.46 | 0.52 | 0.57 | 0.65 | 0.74 | 0.82 | 0.89 |
| Yaxis_Contrac | 0.59 | 0.65 | 0.71 | 0.83 | 0.92 | 1.03 | 1.10 |
| **Men 31-45** | ***5th*** | ***10th*** | ***25th*** | ***50th*** | ***75th*** | ***90th*** | ***95th*** |
| T-SAT/h2 | 0.18 | 0.22 | 0.36 | 0.55 | 0.69 | 0.84 | 0.93 |
| S-SAT/h2 | 0.07 | 0.11 | 0.16 | 0.25 | 0.33 | 0.42 | 0.48 |
| VAT/h2 | 0.08 | 0.10 | 0.15 | 0.22 | 0.30 | 0.40 | 0.50 |
| L-SAT/h2 | 0.10 | 0.12 | 0.16 | 0.21 | 0.30 | 0.38 | 0.43 |
| RF-CSA/h2 | 1.53 | 1.62 | 1.96 | 2.31 | 2.68 | 3.12 | 3.33 |
| RF-CIR/h2 | 2.86 | 3.02 | 3.25 | 3.52 | 3.84 | 4.10 | 4.25 |
| RF-Xaxis/h2 | 1.063 | 1.13 | 1.20 | 1.31 | 1.42 | 1.52 | 1.61 |
| RF-Yaxis/h2 | 0.45 | 0.49 | 0.56 | 0.64 | 0.73 | 0.82 | 0.86 |
| Yaxis_Contrac | 0.62 | 0.65 | 0.73 | 0.82 | 0.93 | 1.01 | 1.06 |
| **Men 46-60** | ***5th*** | ***10th*** | ***25th*** | ***50th*** | ***75th*** | ***90th*** | ***95th*** |
| T-SAT/h2 | 0.29 | 0.35 | 0.45 | 0.60 | 0.76 | 0.93 | 1.02 |
| S-SAT/h2 | 0.14 | 0.15 | 0.20 | 0.27 | 0.34 | 0.42 | 0.46 |
| VAT/h2 | 0.087 | 0.12 | 0.17 | 0.26 | 0.34 | 0.43 | 0.47 |
| L-SAT/h2 | 0.10 | 0.12 | 0.17 | 0.22 | 0.28 | 0.37 | 0.46 |
| RF-CSA/h2 | 1.38 | 1.51 | 1.71 | 2.04 | 2.35 | 2.73 | 3.06 |
| RF-CIR/h2 | 2.77 | 2.90 | 3.20 | 3.45 | 3.76 | 3.96 | 4.17 |
| RF-Xaxis/h2 | 1.04 | 1.11 | 1.22 | 1.33 | 1.43 | 1.54 | 1.58 |
| RF-Yaxis/h2 | 0.40 | 0.44 | 0.51 | 0.58 | 0.66 | 0.74 | 0.79 |
| Yaxis_Contrac | 0.54 | 0.57 | 0.67 | 0.77 | 0.88 | 0.96 | 1.02 |
| **Men >60** | ***5th*** | ***10th*** | ***25th*** | ***50th*** | ***75th*** | ***90th*** | ***95th*** |
| T-SAT/h2 | 0.29 | 0.32 | 0.40 | 0.53 | 0.80 | 0.92 | 1.01 |
| S-SAT/h2 | 0.11 | 0.13 | 0.18 | 0.23 | 0.35 | 0.45 | 0.48 |
| VAT/h2 | 0.09 | 0.12 | 0.14 | 0.22 | 0.27 | 0.38 | 0.48 |
| L-SAT/h2 | 0.10 | 0.12 | 0.16 | 0.22 | 0.27 | 0.34 | 0.43 |
| RF-CSA/h2 | 1.17 | 1.31 | 1.60 | 1.88 | 2.25 | 2.53 | 2.86 |
| RF-CIR/h2 | 2.76 | 2.81 | 3.18 | 3.43 | 3.64 | 4.00 | 4.26 |
| RF-Xaxis/h2 | 1.08 | 1.09 | 1.20 | 1.33 | 1.44 | 1.61 | 1.68 |
| RF-Yaxis/h2 | 0.35 | 0.39 | 0.46 | 0.54 | 0.64 | 0.73 | 0.76 |
| Yaxis_Contrac | 0.44 | 0.53 | 0.62 | 0.73 | 0.84 | 0.94 | 1.00 |

**Supplementary apendix 5 – Percentile tables of NU measurements (cm) between weight (kg) multiplied by 100**

| **Women 18-30** | ***5th*** | ***10th*** | ***25th*** | ***50th*** | ***75th*** | ***90th*** | ***95th*** |
| --- | --- | --- | --- | --- | --- | --- | --- |
| T-SAT, cm/w | 0.95 | 1.18 | 1.67 | 2.38 | 3.08 | 3.54 | 3.92 |
| S-SAT, cm/w | 0.44 | 0.53 | 0.78 | 1.14 | 1.54 | 1.91 | 2.07 |
| VAT, cm/w | 0.35 | 0.44 | 0.62 | 0.88 | 1.11 | 1.55 | 1.69 |
| L-SAT, cm/w | 1.17 | 1.39 | 1.67 | 2.05 | 2.38 | 2.86 | 3.17 |
| RF-CSA, cm2/w | 5.05 | 5.68 | 6.88 | 8.31 | 9.65 | 10.91 | 11.73 |
| RF-CIR, cm/w | 11.31 | 11.96 | 13.44 | 15.07 | 16.80 | 18.35 | 19.12 |
| RF-Xaxis, cm/w | 3.96 | 4.44 | 5.00 | 5.74 | 6.46 | 7.16 | 7.57 |
| RF-Yaxis, cm/w | 1.89 | 2.03 | 2.32 | 2.72 | 3.11 | 3.49 | 3.69 |
| RF-Yaxis_Contrac | 2.48 | 2.62 | 2.97 | 3.40 | 3.90 | 4.35 | 4.52 |
| **Women 31-45** | ***5th*** | ***10th*** | ***25th*** | ***50th*** | ***75th*** | ***90th*** | ***95th*** |
| T-SAT, cm/w | 0.99 | 1.31 | 1.91 | 2.55 | 3.30 | 3.87 | 4.40 |
| S-SAT, cm/w | 0.45 | 0.58 | 0.95 | 1.26 | 1.69 | 2.04 | 2.45 |
| VAT, cm/w | 0.30 | 0.36 | 0.57 | 0.84 | 1.17 | 1.52 | 1.98 |
| L-SAT, cm/w | 1.17 | 1.39 | 1.71 | 2.05 | 2.45 | 2.83 | 3.02 |
| RF-CSA, cm2/w | 4.87 | 5.48 | 6.24 | 7.31 | 8.65 | 10.17 | 11.91 |
| RF-CIR, cm/w | 10.79 | 11.80 | 12.83 | 14.20 | 16.17 | 17.82 | 19.54 |
| RF-Xaxis, cm/w | 4.02 | 4.27 | 4.84 | 5.39 | 6.25 | 7.18 | 7.69 |
| RF-Yaxis, cm/w | 1.78 | 1.95 | 2.18 | 2.50 | 2.80 | 3.12 | 3.37 |
| RF-Yaxis_Contrac | 2.27 | 2.40 | 2.72 | 3.14 | 3.51 | 3.98 | 4.24 |
| **Women 46-60** | ***5th*** | ***10th*** | ***25th*** | ***50th*** | ***75th*** | ***90th*** | ***95th*** |
| T-SAT, cm/w | 1.27 | 1.58 | 2.07 | 2.98 | 3.73 | 4.68 | 4.98 |
| S-SAT, cm/w | 0.54 | 0.73 | 1.00 | 1.45 | 1.91 | 2.35 | 2.67 |
| VAT, cm/w | 0.32 | 0.43 | 0.61 | 0.84 | 1.20 | 1.55 | 1.84 |
| L-SAT, cm/w | 1.16 | 1.41 | 1.67 | 2.09 | 2.50 | 2.94 | 3.31 |
| RF-CSA, cm2/w | 4.34 | 4.60 | 5.42 | 6.48 | 7.59 | 9.09 | 9.93 |
| RF-CIR, cm/w | 9.96 | 10.44 | 11.98 | 13.52 | 15.18 | 16.40 | 17.21 |
| RF-Xaxis, cm/w | 3.74 | 4.04 | 4.60 | 5.21 | 5.98 | 6.49 | 7.06 |
| RF-Yaxis, cm/w | 1.48 | 1.60 | 1.83 | 2.15 | 2.50 | 2.84 | 3.00 |
| RF-Yaxis_Contrac | 1.94 | 2.16 | 2.42 | 2.81 | 3.17 | 3.57 | 3.87 |
| **Women >60** | ***5th*** | ***10th*** | ***25th*** | ***50th*** | ***75th*** | ***90th*** | ***95th*** |
| T-SAT, cm/w | 1.77 | 2.17 | 2.67 | 3.37 | 4.23 | 5.44 | 5.85 |
| S-SAT, cm/w | 0.62 | 0.88 | 1.15 | 1.55 | 2.12 | 2.67 | 3.15 |
| VAT, cm/w | 0.43 | 0.52 | 0.76 | 1.03 | 1.23 | 1.55 | 1.80 |
| L-SAT, cm/w | 1.18 | 1.46 | 1.80 | 2.21 | 2.68 | 3.21 | 3.29 |
| RF-CSA, cm2/w | 3.80 | 4.15 | 4.76 | 5.59 | 6.83 | 8.03 | 9.31 |
| RF-CIR, cm/w | 9.05 | 9.93 | 11.13 | 12.71 | 14.58 | 16.34 | 17.20 |
| RF-Xaxis, cm/w | 3.74 | 4.03 | 4.35 | 5.17 | 5.85 | 6.50 | 7.06 |
| RF-Yaxis, cm/w | 1.24 | 1.37 | 1.63 | 1.96 | 2.38 | 2.61 | 3.09 |
| RF-Yaxis_Contrac | 1.57 | 1.79 | 2.16 | 2.48 | 3.08 | 3.66 | 3.93 |

| **Men 18-30** | ***5th*** | ***10th*** | ***25th*** | ***50th*** | ***75th*** | ***90th*** | ***95th*** |
| --- | --- | --- | --- | --- | --- | --- | --- |
| T-SAT, cm/w | 0.45 | 0.63 | 1.11 | 1.68 | 2.29 | 2.89 | 3.34 |
| S-SAT, cm/w | 0.20 | 0.32 | 0.55 | 0.84 | 1.12 | 1.37 | 1.62 |
| VAT, cm/w | 0.29 | 0.42 | 0.61 | 0.89 | 1.17 | 1.43 | 1.70 |
| L-SAT, cm/w | 0.37 | 0.44 | 0.63 | 0.87 | 1.23 | 1.49 | 1.64 |
| RF-CSA, cm2/w | 6.05 | 6.74 | 7.88 | 9.48 | 10.96 | 12.86 | 13.83 |
| RF-CIR, cm/w | 10.49 | 11.28 | 12.83 | 14.34 | 15.96 | 17.30 | 18.00 |
| RF-Xaxis, cm/w | 3.78 | 4.20 | 4.86 | 5.37 | 6.03 | 6.64 | 6.98 |
| RF-Yaxis, cm/w | 1.86 | 1.99 | 2.28 | 2.63 | 3.03 | 3.34 | 3.54 |
| RF-Yaxis_Contrac | 2.32 | 2.51 | 2.89 | 3.33 | 3.82 | 4.23 | 4.42 |
| **Men 31-45** | ***5th*** | ***10th*** | ***25th*** | ***50th*** | ***75th*** | ***90th*** | ***95th*** |
| T-SAT, cm/w | 0.74 | 0.96 | 1.40 | 2.06 | 2.58 | 3.01 | 3.20 |
| S-SAT, cm/w | 0.30 | 0.42 | 0.64 | 0.93 | 1.24 | 1.54 | 1.77 |
| VAT, cm/w | 0.33 | 0.41 | 0.58 | 0.85 | 1.13 | 1.47 | 1.75 |
| L-SAT, cm/w | 0.38 | 0.46 | 0.62 | 0.82 | 1.10 | 1.36 | 1.61 |
| RF-CSA, cm2/w | 5.82 | 6.32 | 7.46 | 8.68 | 10.19 | 11.70 | 13.06 |
| RF-CIR, cm/w | 10.22 | 10.91 | 12.20 | 13.34 | 15.06 | 16.29 | 17.66 |
| RF-Xaxis, cm/w | 3.67 | 4.12 | 4.46 | 5.06 | 5.63 | 6.15 | 6.65 |
| RF-Yaxis, cm/w | 1.78 | 1.89 | 2.12 | 2.41 | 2.72 | 3.14 | 3.46 |
| RF-Yaxis_Contrac | 2.34 | 2.46 | 2.69 | 3.13 | 3.59 | 3.88 | 4.19 |
| **Men 46-60** | ***5th*** | ***10th*** | ***25th*** | ***50th*** | ***75th*** | ***90th*** | ***95th*** |
| T-SAT, cm/w | 1.07 | 1.27 | 1.71 | 2.20 | 2.70 | 3.17 | 3.44 |
| S-SAT, cm/w | 0.48 | 0.58 | 0.75 | 0.96 | 1.24 | 1.49 | 1.67 |
| VAT, cm/w | 0.31 | 0.42 | 0.62 | 0.91 | 1.23 | 1.57 | 1.77 |
| L-SAT, cm/w | 0.35 | 0.46 | 0.62 | 0.81 | 1.02 | 1.28 | 1.53 |
| RF-CSA, cm2/w | 5.19 | 5.45 | 6.40 | 7.41 | 8.73 | 10.10 | 10.96 |
| RF-CIR, cm/w | 9.83 | 10.43 | 11.26 | 12.56 | 14.06 | 15.14 | 15.82 |
| RF-Xaxis, cm/w | 3.68 | 3.86 | 4.29 | 4.77 | 5.46 | 5.86 | 6.14 |
| RF-Yaxis, cm/w | 1.52 | 1.63 | 1.83 | 2.11 | 2.43 | 2.71 | 2.86 |
| RF-Yaxis_Contrac | 1.90 | 2.11 | 2.41 | 2.82 | 3.17 | 3.58 | 3.86 |
| **Men >60** | ***5th*** | ***10th*** | ***25th*** | ***50th*** | ***75th*** | ***90th*** | ***95th*** |
| T-SAT, cm/w | 0.96 | 1.13 | 1.44 | 2.08 | 2.72 | 3.34 | 3.72 |
| S-SAT, cm/w | 0.36 | 0.46 | 0.64 | 0.91 | 1.25 | 1.54 | 1.63 |
| VAT, cm/w | 0.29 | 0.42 | 0.55 | 0.79 | 1.04 | 1.35 | 1.74 |
| L-SAT, cm/w | 0.39 | 0.47 | 0.61 | 0.76 | 0.94 | 1.18 | 1.35 |
| RF-CSA, cm2/w | 4.67 | 4.92 | 5.67 | 6.59 | 8.67 | 9.55 | 10.00 |
| RF-CIR, cm/w | 9.50 | 10.19 | 10.85 | 12.51 | 14.13 | 15.25 | 15.83 |
| RF-Xaxis, cm/w | 3.43 | 3.74 | 4.15 | 4.82 | 5.56 | 5.96 | 6.08 |
| RF-Yaxis, cm/w | 1.32 | 1.43 | 1.69 | 1.95 | 2.37 | 2.60 | 2.73 |
| RF-Yaxis_Contrac | 1.68 | 1.79 | 2.27 | 2.61 | 3.06 | 3.53 | 3.70 |
